# Assessing the impact of geographical access to health facilities on maternal healthcare utilization: evidence from the Burkina Faso demographic and health survey 2010

**DOI:** 10.1186/s12889-019-7150-1

**Published:** 2019-06-27

**Authors:** Mariam Tanou, Yusuke Kamiya

**Affiliations:** 1 Ministry of Infrastructure, Building Lamizana, Ouagadougou, 03BP7011 Burkina Faso; 2 Ryukoku University, Faculty of Economics, 67 Tsukamoto-cho, Fukakusa, Fushimi-ku, Kyoto, 612-8577 Japan

**Keywords:** Geographical access, Maternal healthcare, Antenatal care, Skilled birth attendant, Geographic information system, Burkina Faso

## Abstract

**Background:**

Improving maternal and child health (MCH) remains a serious challenge for many developing countries. Geographical accessibility from a residence to the nearest health facility is suspected to be an important obstacle hampering the use of appropriate services for MCH especially in Sub-Sharan African countries. In Burkina Faso, a landlocked country in the Sahel region of West Africa, women’s use of proper healthcare services during pregnancy and childbirth is still low. This study therefore assessed the impact of geographical access to health facilities on maternal healthcare utilization in Burkina Faso.

**Methods:**

We used the Burkina Faso demographic and health survey (DHS) 2010 dataset, with its sample of 10,364 mothers aged 15–49 years. Distance from residential areas to the closest health facility was measured by merging the DHS dataset with Geographic Information System data on the location of health centers in Burkina Faso. Multivariate logistic regressions were conducted to estimate the effects of distance on maternal healthcare utilization.

**Results:**

Regression results revealed that the longer the distance to the closest health center, the less likely it is that a woman will receive appropriate maternal healthcare services. The estimates show that one kilometer increase in distance to the closest health center reduces the odds that a woman will receive four or more antenatal care by 0.05 and reduces by 0.267 the odds that she will deliver her baby with the assistance of a skilled birth attendant.

**Conclusions:**

Improving geographical access to health facilities increases the use of appropriate healthcare services during pregnancy and childbirth. Investment in transport infrastructure should be a prioritized target for further improvement in MCH in Burkina Faso.

## Background

Maternal and child mortality rates in the Sahel region of West Africa are among the highest in the world [1]. It has been confirmed that a woman’s use of proper healthcare services during pregnancy and childbirth has positive impacts on both maternal and child survival [2, 3]. It seems clear that improving maternal and child health (MCH) is vital to any country’s long-term development [4] and that more efforts should be made to promote the use of essential healthcare during pregnancy and childbirth.

In Burkina Faso, a landlocked country in West Africa, the maternal mortality ratio declined from 727 to 371 per 100,000 livebirths between 1990 and 2015, a decrease of 49% over this period [1]. Despite this downward trend in maternal mortality, MCH remains a major challenge. Although the percentage of women who visited a healthcare facility for at least one antenatal care (ANC) was 95% in 2010, only 34% received the recommended number of at least four ANC [5].

Demographic, socioeconomic and environmental factors have long been confirmed to be important determinants of MCH [6–9]. In Sub-Saharan Africa, in particular, geographical accessibility to health facilities is considered a major obstacle to the improvement of maternal healthcare utilization [10–14]. The relationship between longer distances to health facilities and low healthcare utilization is often called the “distance decay” effect, and this effect has been observed in many countries in Sub-Saharan Africa [15–18], as well as Asia [19, 20].

Several related studies have been conducted in Burkina Faso [21–25]. One study, in the Ouargaye district, a rural area located 225 km southeast of Ouagadougou (the capital of Burkina Faso), confirmed that distance to a health facility was a major determinant of institutional birth-delivery with skilled care [21]. In other study, living within 5 km from a health facility was associated with increased uptake of ANC and institutional birth-delivery in the Nouna Health District in north-western Burkina Faso [22]. However, no study appears to have analyzed the effects of geographical access to health facilities on maternal healthcare utilization at the national level in Burkina Faso. In addition, empirical studies using nationally representative data in the Sahel region in West Africa, where geographical access to health facilities is particularly important, are quite limited [26]. We therefore conducted our study to analyze geographical access to health facilities on maternal healthcare utilization in Burkina Faso. Among several important implications, our findings can be expected to help policymakers better understand the role of transport infrastructure in the improvement of MCH in Sub-Sharan Africa.

## Methods

### Data

The demographic and health survey (DHS) of Burkina Faso 2010 was used for the empirical analysis in our study [27]. The DHS is a nationally representative survey that applies a stratified two-stage cluster sampling design. The sample is stratified into urban and rural areas to represent both areas. In the sampling, the primary survey units (“clusters”) are first selected from larger 13 regional units based on 2006 General Population and Housing Census and then individual households are randomly selected within each cluster. The data were collected between May 2010 and January 2011. In all, 14,947 households from 574 clusters (398 from rural areas and 176 from urban areas) were sampled, and then 14,424 households were actually surveyed. (The response rate was 99.2%). The study population for our analysis was 10,364 women (15–49 years old) who lived in these households and had a live birth in the 5 years preceding the survey [27].

Our main explanatory variable was distance from a residential area to the closest health center. Because the DHS household questionnaires contained no information regarding distance or travel time to health facilities, we used the Geographical Information System (GIS) module of the DHS to calculate these distances. We also obtained geographic information on roads and health centers through the Ministry of Infrastructure of Burkina Faso (for road information) and the Centre National de Recherche Scientifique et Technologique of Burkina Faso (for health center information).

For measuring the distance between a residence and the closest health facility, a previous study conducted in Ghana employed the following methods [28]: 1) Euclidean distance (km), the straight-line distance from a residence to the closest health facility; 2) network distance (km), the distance along the road network from a residence to the closest health facility plus the Euclidean distance to the road network from a residence and from the road network to the health facility; 3) network travel time (hour), the distance along the road network from a residence to the closet health facility plus the Euclidean distance multiplied by off-road walking speed to the road network from the residence and from the road network to the health facility; and 4) raster-based travel time (hour), the travel time from a residence to the closest health facility, assuming mechanized or non-mechanized travel on roads and non-mechanized travel on- or off-road depending on the land cover speed [28]. Results obtained by these methods were similar in Ghana [28]. We chose the Euclidean distance to measure the distance from a cluster centroid to the closest health center and then used it as a proxy for the geographical access to a health center from a residential area. The advantage of this method is that it can be generalized for other similar topography and cultural contexts in West Africa [28].

**Fig. 1:**
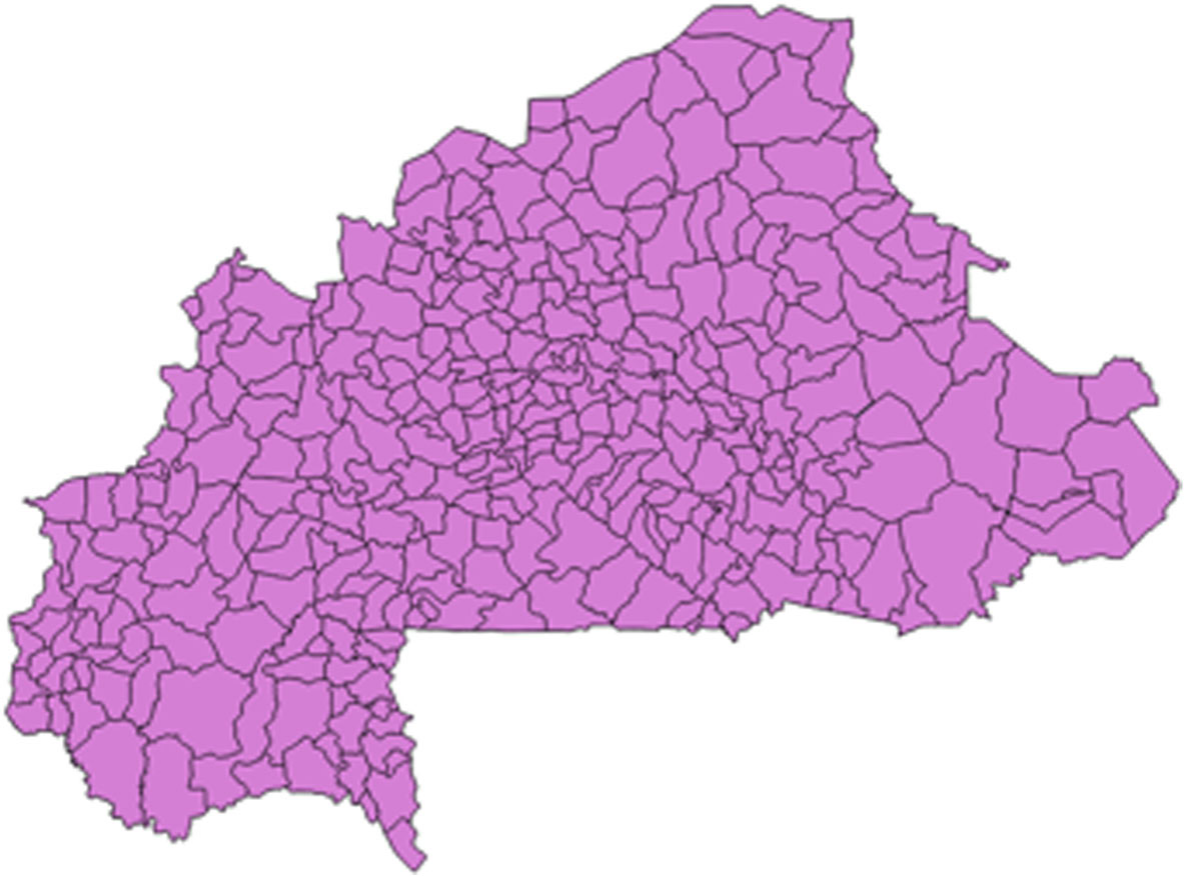
Burkina Faso divided by communes

**Fig. 2:**
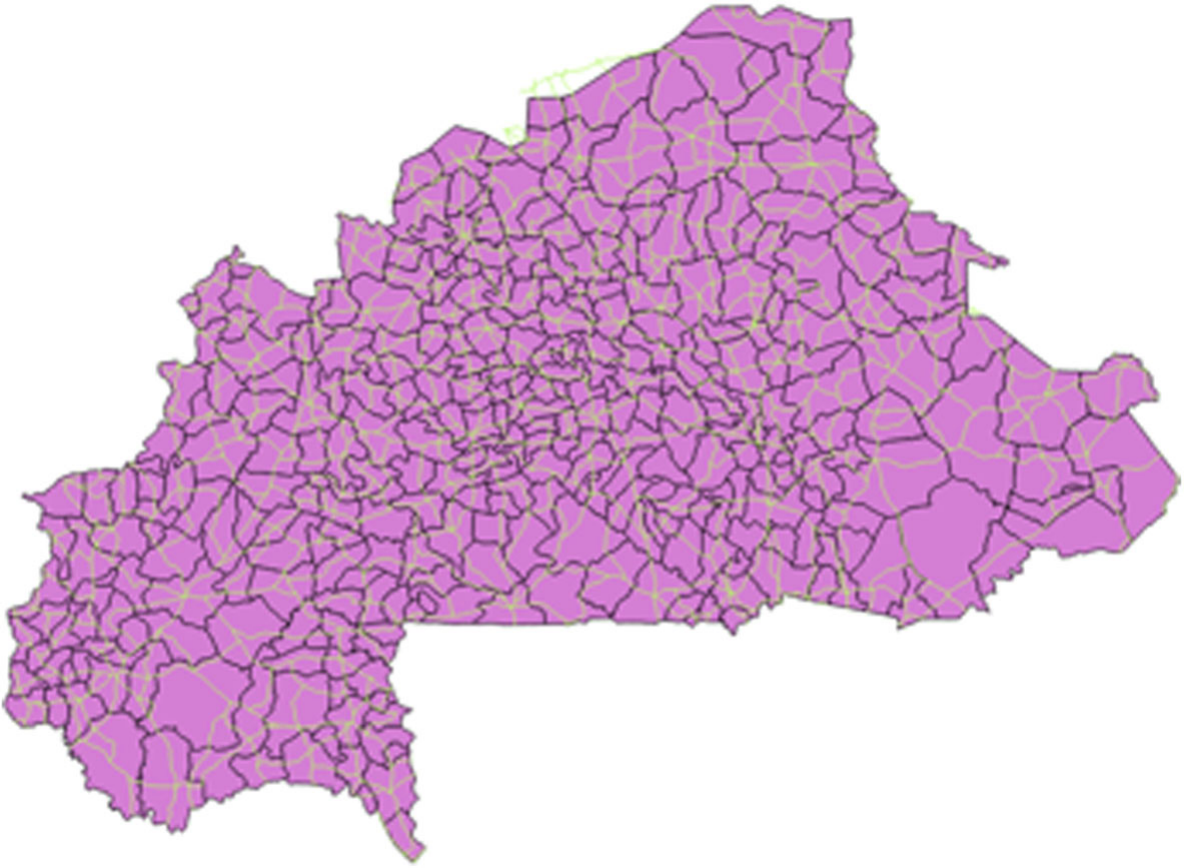
Road network

**Fig. 3:**
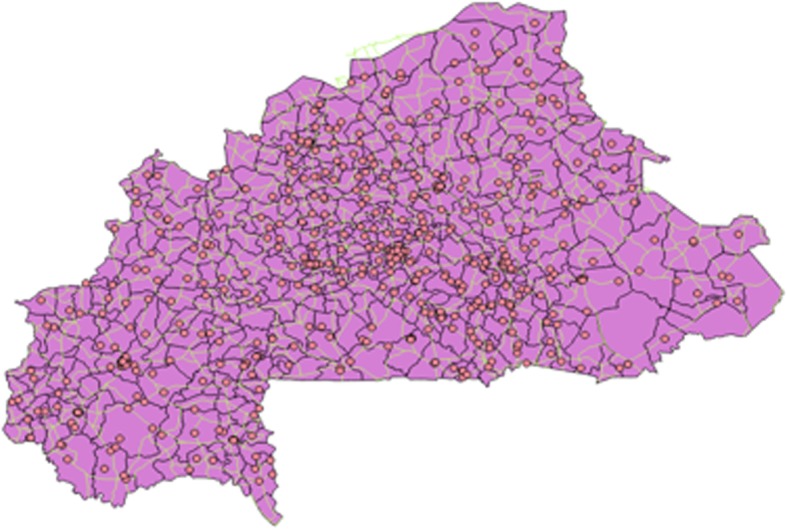
GIS points of clusters

**Fig. 4:**
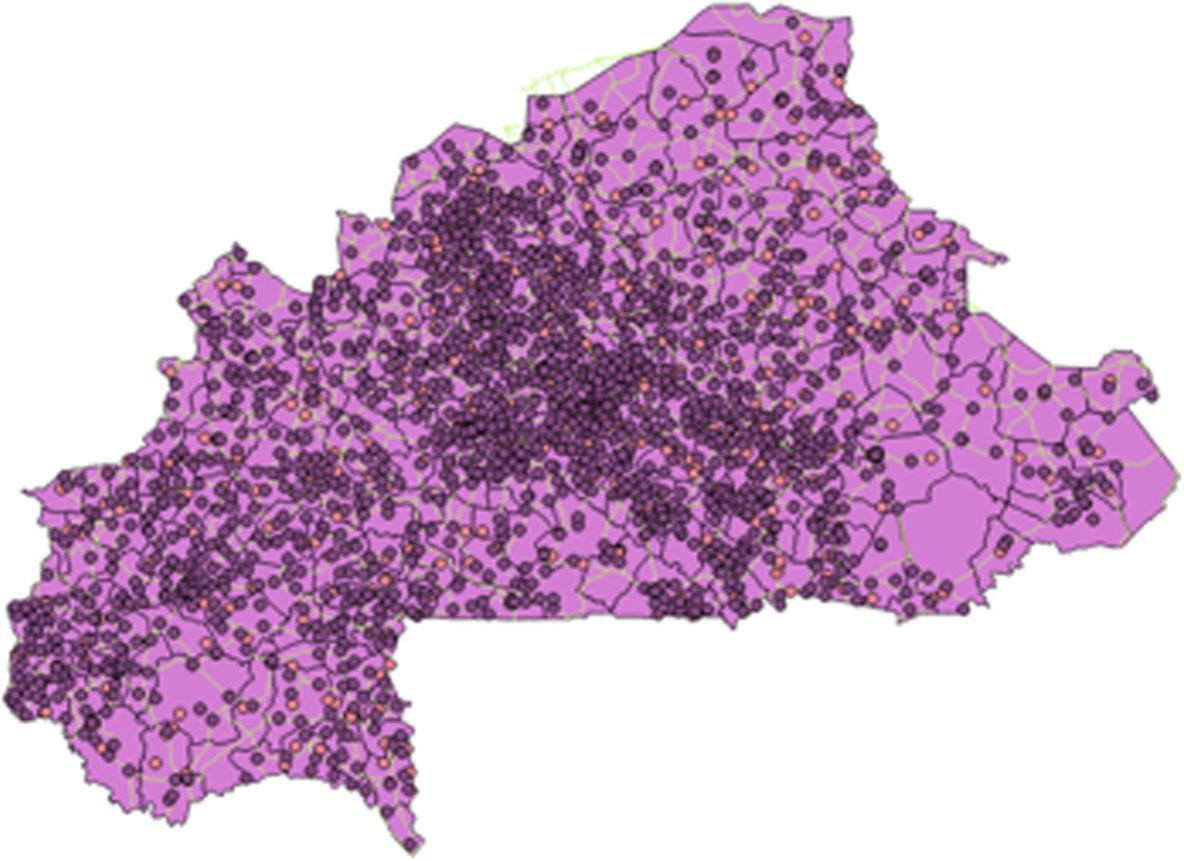
GIS points of health centers

Figure 1 shows a map of Burkina Faso, divided into 352 communes. (Burkina Faso is divided into 13 administrative regions, which are subdivided into 45 provinces; the provinces are subdivided into 352 communes) Fig. 2 shows the country’s network of roads. In Fig. 3, the red circles indicate the 574 clusters from which households were randomly selected for the survey. Figure 4 shows the location of 1520 health centers (purple circles). We did not distinguish the level of health services provided by each center. Finally, we used Quantum-GIS software to calculate the Euclidean distance (km) from each cluster to the closest health center.

In addition to the distance to health facilities, we analyzed if the availability of means of transport at the community-level was associated with the use of maternal healthcare. DHS did not include information on public transport, but asked questions about whether or not the household owned a bicycle and motorbike, which are the popular means of transport in Burkina Faso even among women in seeking healthcare during pregnancy and childbirth [23]. We thus calculated the ownership rates of bicycles and motorbikes per cluster and used them as proxy variables for the community-level availability of means of transport.

### Statistical analysis

Multivariate logistic regressions were conducted to analyze the effect of distance to the closest health center on maternal healthcare utilization. Data analysis was carried out using Stata 14.0. Because DHS applied a two-stage cluster sampling design, we used the svy (survey) commands of Stata to correct for unequal sampling probability, clustering and stratification in calculating descriptive statistics and performing regression analysis.

### Outcome variables

We used the following outcome variables: 1) whether the woman made at least one ANC visit during her latest pregnancy (“Received any ANC”); 2) whether the woman made four or more ANC visits during her latest pregnancy (“Received ≥ 4 ANC”); 3) whether the woman used a health facility at the birth-delivery (“Facility delivery”); and 4) whether the woman was attended by a professional health worker, i.e., doctor, nurse, auxiliary nurse or midwife at the birth-delivery (“Delivery by SBA” (skilled birth attendant)). Because all the outcome variables were binary, they were coded 1 if the mother had received appropriate healthcare (ANC, Facility delivery, or SBA) during her pregnancy or at childbirth, or 0 otherwise. Outcome variables for birth-delivery, i.e. 3) “Facility delivery” and 4) "Delivery by SBA, included all the births (14,996) that had taken place during the five years preceding the survey. Therefore, we included mother-level random intercepts into multivariate logistic regressions for 3) “Facility delivery” and 4) "Delivery by SBA to adjust for the correlation of births to the same mother.

### Control variables

We used demographic and socioeconomic characteristics at the mother, household, and community levels as control variables. The mother-level variables consisted of age and educational achievement (no education, primary, secondary, and higher). The household-level variables included the religion of the household head (no religion, Muslim, Catholic, Protestant, and traditional religion/animist), asset quintiles. The community-level variables included area dummies (rural or urban). In addition, region dummies (Boucle du Mouhoun, Cascades, Centre, Centre-Est, Centre-Ouest, Centre-Nord, Centre-Sud, Est, Hauts Basins, Nord, Plateau Central, Sahel, and Sud-Ouest) were included to consider regional differences within the country.

## Results

### Sample characteristics

Table 1 summarizes characteristics of the sample. The data was corrected for a cluster survey design by calculating sampling weights which were available in the DHS dataset. In all, 10,364 mothers and 14,996 births were included in the study. As shown, 94.8% of the women received ANC at least once, whereas only 33.6% received ANC four or more times; 66.7% delivered their babies at a health facility; and 67.4% were assisted by an SBA during childbirth. The mean distance to the closest heath center, the main predictor in the regression, was 4.47 km.

**Table 1:** Sample characteristics

Variables	N	Mean	S.D.
Mothers’ outcome variables			
Received any ANC	10,356	0.948	0.223
Received ≥4 ANC	10,356	0.336	0.472
Facility delivery	14,970	0.667	0.471
Delivery by SBA	14,996	0.674	0.469
Main predictor			
Distance to the closest health center (km)	9764	4.47	3.67
Mothers’ characteristics			
Age	10,364	29.3	7.18
Education			
No formal education	10,361	0.824	0.380
Primary	10,361	0.114	0.318
Secondary/Higher	10,361	0.061	0.240
Households’ characteristics			
Religion			
Muslim	10,333	0.637	0.481
Catholic	10,333	0.211	0.408
Protestant	10,333	0.062	0.241
Traditional/ Animist	10,333	0.081	0.272
No religion	10,333	0.010	0.098
Asset			
Lowest	10,364	0.194	0.396
Lower middle	10,364	0.210	0.407
Middle	10,364	0.213	0.410
Upper middle	10,364	0.211	0.408
Highest	10,364	0.172	0.377
Community’s characteristics			
Area			
Rural	10,364	0.809	0.393
Urban	10,364	0.191	0.393
Ownership rate			
Bicycle	10,364	0.895	0.160
Motorbike	10,364	0.393	0.218

Unweighted N, and weighted mean and S.D. are reported

The mean age of the mothers was 29.3 years. Majority of the mothers (82.4%) had not received formal education; only 6.1% had finished secondary education or higher. As for household characteristics, 63.7% of the household heads were Muslim, followed by Catholic (21.1%), traditional/ animist (8.1%) and Protestant (6.2%). Regarding community’s characteristics, 80.9% of the households were in rural areas. Mean of the community-level ownership rate of bicycle and motorbike was 89.5 and 39.3%, respectively.

### Regression analysis

Table 2 presents results from the multivariate logistic regressions for maternal health care utilization. The magnitude of effects was assessed by odds ratios (ORs), which can be interpreted as increasing the likelihood of using maternal healthcare (if OR > 1) or reducing that likelihood (if OR < 1). With respect to the main predictor—distance to the closest health center—there was a statistically significant and negative effect on all four maternal healthcare utilization variables, indicating that the longer the distance to the closest health center, the less likely women are to receive the necessary healthcare during pregnancy and childbirth. The estimated ORs for “Received any ANC” (OR = 0.887, *p* < 0.001) and “Received ≥ 4 ANC” (OR = 0.950, *p* < 0.001) indicate that if distance to the closest health center increases by one kilometer, the odds of a woman receiving ANC at least once is reduced by 0.113, and the odds of a woman receiving ANC at least four times declines by 0.05. Similarly, the estimated ORs for “Facility delivery” (OR = 0.726, *p* < 0.001) and “Delivery by SBA” (OR = 0.733, *p* < 0.001) suggest that one kilometer increase in distance decreases the odds of delivering a baby at a health facility by 0.274 and reduces the odds of being assisted by an SBA by 0.267.

**Table 2:** Results of multivariate logistics regressions for maternal healthcare utilization

	Received any ANC	Received ≥4 ANC	Facility delivery	Delivery by SBA
Main predictor				
Distance to a health facility	0.887 (0.000)***	0.950 (0.000)***	0.726 (0.000)***	0.733 (0.000)***
Mother-level				
Age	0.963 (0.000)***	0.994 (0.080)	0.962 (0.000)***	0.963 (0.000)***
Education				
No education †				
Primary	2.778 (0.000)***	1.235 (0.005)**	3.926 (0.000)***	3.975 (0.000)***
Secondary/Higher	1.931 (0.256)	1.950 (0.000)***	16.826 (0.000)***	17.918 (0.000)***
Household-level				
Religion				
Muslim †				
Catholic	1.378 (0.118)	1.103 (0.141)	2.110 (0.001)***	2.345 (0.000)***
Protestant	1.327 (0.366)	1.574 (0.000)***	3.246 (0.000)***	2.652 (0.002)**
Traditional/ Animist	0.514 (0.004)**	0.737 (0.044)*	0.377 (0.004)**	0.360 (0.004)**
No religion	0.324 (0.013)*	0.947 (0.816)	0.913 (0.853)	0.912 (0.857)
Asset				
Lowest †				
Lower middle	1.250 (0.131)	1.171 (0.077)	1.523 (0.004)**	1.677 (0.001)***
Middle	1.255 (0.175)	1.258 (0.013)*	2.141 (0.000)***	2.418 (0.000)***
Upper middle	2.718 (0.000)***	1.541 (0.000)***	4.122 (0.000)***	4.428 (0.000)***
Highest	7.243 (0.000)***	1.758 (0.000)***	8.694 (0.000)***	9.542 (0.000)***
Community-level				
Area				
Rural †				
Urban	0.863 (0.545)	0.880 (0.206)	3.761 (0.000)***	3.825 (0.000)***
Ownership rate				
Bicycle	8.379 (0.001)***	1.085 (0.810)	11.856 (0.008)**	9.867 (0.017)*
Motorbike	0.796 (0.638)	0.748 (0.164)	0.720 (0.625)	0.898 (0.877)
Observation	9726	9726	14,047	14,074

Odds ratios (ORs) are reported, *p < 0.05 **p < 0.01 ***p < 0.001,

†Reference, Region dummies were also included as control variables

Finally, with respect to the other explanatory variables at the individual and household levels, the mother’s educational attainment and the wealth of the household were rather consistently associated with a higher likelihood of ANC use, facility-based delivery and SBA support. Regarding religion, women whose head of household believed in a traditional religion/animist were less likely to receive maternal healthcare than those from Muslim households. In contrast, women from Catholic and Protestant households were more likely to use a health facility and SBA for birth-delivery. As for the community-level variables, women living in urban area were more likely to use facility-based delivery and SBA than those in rural area. Looking at the availability of means of transport, the ownership rate for bicycles was associated with a higher likelihood of women’s use of ANC at least once, facility-based delivery and SBA support.

## Discussion

We examined the effects of the distance to health facilities on the use of maternal healthcare services in Burkina Faso by analyzing a national-representative sample from the DHS dataset along with the GIS data on health center locations across the country. We confirmed that a distance from a residential area to the closest health center, after controlling for potential confounders, had a significantly negative impact on the use of appropriate healthcare for pregnancy and childbirth.

The results showing a negative effect of the longer distance on utilization of maternal healthcare are consistent with those of previous studies in Burkina Faso [21–25]. While these past studies were conducted in limited rural areas, our study confirmed a significant and negative effect of distance at the national level in Burkina Faso. The result is also in line with previous studies conducted at the national level in other Sub-Saharan African countries including Ghana, Malawi and Zambia [10–14]. Since empirical evidence obtained from a nationally representative sample in the Sahel region of West Africa, where geographical access to health facilities is particularly important, is quite limited [26], our study will be an important benchmark for the future studies and policy orientation.

In addition to the distance, we confirmed that the availability of means of transport at the community-level proxied by the bicycle ownership rate was associated with a higher likelihood of the use of maternal healthcare. In contrast, the motorbike ownership rate was not a statistically significant predictor of maternal healthcare utilization. It indicates that bicycles are more common as the means of transport compared to motorbikes among women seeking healthcare during pregnancy and childbirth in Burkina Faso. On this topic, a study conducted in north-western Burkina Faso showed a positive correlation between the availability of a motorbike in the woman’s household and their use of a health facility for delivery [23]. Further studies are needed to explore how a mode of transport correlates with the utilization of maternal healthcare in Burkina Faso.

Regarding mothers’ individual characteristics, educational attainment positively correlated with the use of maternal healthcare. Since empirical results obtained from past studies on this topic in rural Burkina Faso are mixed [21–23, 25], our results obtained from a national-representative data would be an important contribution. Regarding religion, when the head of a household believed in a traditional religion or animist, women in the household were less likely to receive appropriate maternal healthcare compared to women from Muslim households. Further, women from Catholic and Protestant households were more likely to have a childbirth at the facility and use SBA. Similar results were obtained from a study in north-western Burkina Faso [22] as well as from a national-representative sample in Ghana [29]. Regarding the economic status of the households, in line with several previous studies in Burkina Faso [21, 25], asset level was confirmed to be a significant and positive predictor of maternal healthcare utilization. Finally, regarding the type of residential area, women living in urban areas were more likely to use health facility and SBA for childbirth than those in rural areas. Nevertheless, there were no significant differences between rural and urban areas in receiving ANC, suggesting there are no big obstacles for women in receiving ANC in rural areas in Burkina Faso.

Our research was not without limitations. Causal relationship between distance to health facility and the MCH variables was not fully confirmed due to the nature of the cross-sectional data. Nevertheless, reverse causality, i.e., families who care more about MCH prefer to live closer to health centers, is not realistic; causality from distance to the MCH variables is much more plausible.

## Conclusions

This study demonstrated that distance to the closest health center, after controlling for other factors, is an important predictor of a woman’s use of appropriate healthcare during pregnancy and childbirth in Burkina Faso. This result suggests that investment in transport infrastructure should be a prioritized target for further improvement in MCH.

## Data Availability

The dataset used during the current study are in the public domain and can be obtained from the DHS Program (http://dhsprogram.com/) or from the corresponding author on reasonable request.

## Abbreviations

ANC: Antenatal care
DHS: Demographic and health survey
GIS: Geographic Information System
MCH: Maternal and child health
SBA: Skilled birth attendant
